# Exploration of Stereoselectivity in Embryo-Larvae (*Danio rerio*) Induced by Chiral PCB149 at the Bioconcentration and Gene Expression Levels

**DOI:** 10.1371/journal.pone.0155263

**Published:** 2016-05-09

**Authors:** Tingting Chai, Feng Cui, Xiyan Mu, Yang Yang, Chengju Wang, Jing Qiu

**Affiliations:** 1 College of Science, China Agricultural University, Beijing, China; 2 Institute of Quality Standards & Testing Technology for Agro-Products, Key Laboratory of Agro-product Quality and Safety, Chinese Academy of Agricultural Sciences, Key Laboratory of Agri-food Quality and Safety, Ministry of Agriculture, Beijing, China; 3 Center of Fishery Resources and Ecology Environment Research, Chinese Academy of Fishery Sciences, Beijing, China; The University of Iowa, UNITED STATES

## Abstract

This paper was designed to study stereoselective enrichment and changes in gene expression when zebrafish (*Danio rerio*) embryo-larvae were exposed to racemic, (-)- or (+)- PCB149 (2,2’,3,4’,5’,6- hexachlorobiphenyl). Based on bioconcentration analysis, non-racemic enrichment was significantly observed after racemic exposure. No isomerization between the two isomers was found after (-)/(+)-PCB149 exposure. Furthermore, based on gene expression-data mining, CYPs genes (*cyp2k6*, *cyp19a1b*, and *cyp2aa4*) were differential genes after (+)-PCB149 exposure. No obvious differences of dysregulation of gene expression caused by racemic and (-)-PCB149, were observed in embryo-larvae. The above results suggested that (-)-PCB149 could be considered as the main factor causing the dysregulation of gene expression in embryo-larvae after racemic exposure; and (+)-PCB149 should be pursued apart from the racemate, when considering the toxicity of chiral PCB149. Thus, the information in our study could provide new insights to assess the environmental risk of chiral PCBs in aquatic systems.

## Introduction

Although polychlorinated biphenyls (PCBs) have been banned since the mid-1970s, these chemicals are still considered as ubiquitous environmentally persistent organic pollutants (POPs) [1]. PCBs are chemically and thermally stable and, therefore, persist in the environment. Previous studies have demonstrated that PCBs have toxicological impacts [2] such as endocrine, and immune system effects in PCBs-contaminated areas [3,4]. It is still essential to trace their environmental fate and potential adverse effects.

A group of 19 PCB congeners contains a chiral axis and exists as two stable rotational atropisomers [5]. No atropisomeric enrichment happens in physico-chemical processes, but atropisomeric processes can be observed in biological processes such as biotransformation [6] and uptake from food or particles [7]. The stereoselective distributions of chiral PCBs have been reported in environmental and biota samples in previous studies [8]. Inferences about stereoselectively biological processes could be derived from toxicological studies [9]. However, little reports were about the toxicology of PCB atropisomers. For example, PCB84 atropisomers atropselectively had neurodevelopmental toxicity on rat cerebellar [10]. (-)-PCB136 enhanced the binding of [^3^H] ryanodine to high-affinity sites on ryanodine receptors type 1 and type 2, whereas (+)-PCB136 is inactivated, which suggested that stereoselectively toxicological impacts on Ca^2+^ channel [11]. Thus, the extent of stereoselective toxicology of chiral PCBs is essential to be observed.

Fish represent an important source of human PCB exposure. Fish may expose to PCBs through water, suspended particles, sediment and diet [8]. Most previous studies showed racemic existence of chiral PCB149 in groupers (*Epinephelus marginatus*) collected between 1994 and 1995 from the northwest African Atlantic Ocean [12]. This has also been reported in Arctic cod (*Boreogadus saida*) [13]. PCB149 was also found to be racemic in the juvenile rainbow trout [14] and in Arctic char (*Salvelinus alpinus*) [15] in laboratory experiments. However, few previous studies have found non-racemic existence such as that observed in Lake Superior trout [16]. The reasons for non-racemic or racemic PCB149 chiral signatures are still unclear and further studies are required to gain insight into chiral PCB149 in fish species.

Embryonic zebrafish have been employed as a model organism for toxicological studies, especially in the field of genetics [17,18]. For fish, the lipid normalized concentration ratio during embryo-to-adult transformation can be considered to assess the adult transfer potential of chemicals in oviparous organisms [19]. Zebrafish embryos are sensitive to environmental changes [20], easy to maintain and handle, and undergo rapid development [21]. Thus, it is important to investigate stereoselective toxicological properties of chiral PCBs in embryonic zebrafish. In addition, new molecular approaches allow for more insight to be gained into toxicological injury. Changes in mRNA transcript levels represent a key component of the biological response of an animal to chemical contaminant exposure [22]. For instance, changes in gene expression have been associated with exposure to PCBs in Arctic beluga whales (*Delphinapterus leucas*) [23], killer whales (*Orcinus orca*) from the northwest Pacific Ocean [22], and free-ranging harbor seals (*Phoca vitulina*) [24].

In our study, bioconcentration in embryo-larvae was analyzed over increasing time and concentration after zebrafish were exposed to the racemate and the isomers of chiral PCB149. The purpose was to explore whether there was stereoselective enrichment of the single isomers. In addition, changes in gene transcription related to oxidative stress and metabolic pathway, were also investigated, to evaluate any stereoselective effects at the mRNA level. The correlation between the bioconcentration, and gene transcription was studied through statistical analysis. This information is intended to provide new insights into the assessment of the environmental risk of chiral PCBs in aquatic systems.

## Materials and Methods

### Zebrafish Husbandry

Juvenile AB strain zebrafish (*Danio rerio*) were obtained from Beijing Hongdagaofeng Aquarium Department and cultured in the fish facility (Esen Corp.) at 26°C with a photoperiod of 14/10 (light/dark) [25]. The adult zebrafish were fed dried brine shrimp (equivalent to 2% of the fish body weight) daily. Healthy developing embryos were identified under a microscope within 2h of natural spawning of healthy adults, and grown in embryo medium.

### Chemicals and Reagents

Racemic PCB149 (99.9%) containing 50% of (-)-PCB149 and 50% of (+)-PCB149 was provided by Dr. Ehrenstorfer GmbH (Germany). The racemate was separated and prepared on a Lux Cellulose-2 column (250 × 4.6 mm, 5 μm, Phenomenex, Torrance, CA) using an Agilent 1200 series high performance liquid chromatography (HPLC) instrument (Wilmington, DE). The mobile phase was 100% *n*-hexane at a flow rate of 1.0 mL/ min. The atropisomers (-)-PCB149 and (+)-PCB149[26] were repeatedly collected separately and concentrated to dryness using a nitrogen-evaporator (Hangzhou Allsheng Instruments company, China) and then dissolved in acetone (Fisher Scientific, USA). The purities and concentrations of the isomers were determined using a gas chromatography-mass spectrometry (GC-MS). Both of the (-) and (+) PCB149 atropisomers were pure (>98.0%).

The reconstituted water also considered as standard solution for embryo-larvae was prepared in the lab with the formula of iso-7346-3, which contained 2 mM Ca^2+^, 0.5 mM Mg^2+^, 0.75 mM Na^+^ and 0.074 mM K^+^ (ISO, 1996) were used for following tests. All organic reagents in this study were of HPLC-grade and the other reagents of analytical grade.

### Exposure and sample collection

This study was performed in conformity with Chinese legislation and approved by the independent animal ethics committee at China Agricultural University. During exposure experiments, surroundings including temperature, humidity and light cycle were the same as the culture environment.

At 3 hour-post-fertilization, 80 healthy embryos were chosen randomly and transferred into 1L glass beakers containing 400mL of test solution. Test solutions were made up in triplicate for each exposure level, and contained the indicated concentrations of racemate, or (-)-PCB149, or (+)-PCB149 in standard solution. The concentration of acetone in the test solutions was less than 0.01% (v/v). Each exposure for embryo-larvae contained a dose of 0 ng/L, 0.5 ng/L, 0.1 μg/L, or 2.5 μg/L in 400ml. Three levels of exposure were designed according to the aquatic environment (0.5 ng/L and 2.5 μg/L) [27] and the maximum permitted level (0.1μg/L) for individual PCB compound in drinking water [28]. This process was replicated, replacing racemate with either the (-)- or (+)- PCB149. 20 embryos or larvae from each treatment in triplicate were collected at 3, 7, 11 day-post-exposure (dpe) for concentration analysis and gene transcription. The exposure time was set according to the hatching point (3d), yolk sac disappearing point (7d) and death point without food (11d). During the exposure period, few dead embryo because of improper handling were removed immediately. Exposure medium was renewed every 24h. Samples for gene transcription were kept overnight in RNA storage solvent (Tiangen Biotech, China) at 4°C, and then stored at -80°C for RNA extraction without RNA storage solvent.

### Determination of PCB in water and sample

During the embryo-larvae exposure test, solutions were collected for determination of PCB atropisomers at the beginning of treatment and 24 h post exposure. Water samples (1 L) were subjected to five liquid-liquid extractions with *n*-hexane (100 mL each time) in a separator funnel along with violent shaking. The *n*-hexane layer was transferred to heart-shaped flasks and concentrated to near-dryness by rotary evaporation (Shanghai Ailang Instruments, Shanghai, China) at 35°C. The concentrated solutions were then blown to dryness by nitrogen evaporation and the residue was again dissolved in 0.1 mL of isooctane for GC-MS analysis.

Twenty embryos or larvae samples in triplicate per treatment were weighed and homogenized with 0.1 mL isooctane using an electric homogenizer (Tiangen Biotech, China). After 20min ultrasonic extraction, samples were centrifuged at 5000 rpm for 10 min. The supernatant after 0.22-μm fiter was for GC-MS analysis.

An Agilent 7890A/5975C GC-MS system equipped with a Chirasil-Dex capillary column (25 m × 0.25 mm; I.D. 0.25 μm df) from Agilent was used for the purity and concentration determinations. The oven temperature was programmed as follows: 60°C for 2 min, 60–150°C at 10°C·min^-1^ (held for 5 min), 150–180°C at 1°C·min^-1^ (held for 22 min). SIM ions were m/z 360 (quantification ion), 362, and 358 [29].

### Gene expression studies

Total RNA was extracted from embryo-larvae by RNAprep Pure Tissue Kit (Tiangen Biotech, China). RNA quality was determined from the quality of 28s and 18s rRNA following 2% agarose gel electrophoresis. The purity was assessed based on the ratio of OD_260_/OD_280_ and the concentration was determined by OD_260_ by UV1240 spectrophotometer (Perkin Elmer, USA). First-strand complementary DNA (cDNA) was synthesized from 0.5 μg of total RNA using FastQuant RT Kit (Tiangen Biotech, China).

Quantitative real-time polymerase chain reaction (qPCR) was performed using SuperReal PreMix Plus Kit (Tiangen Biotech, China) and measured with an ABI 7500 q-PCR system (Applied Biosystems, USA). The primer sequences were designed with Primer 6.0, and are shown in Table 1. The house-keeping gene β-actin, was used as an internal standard to eliminate variations in mRNA and cDNA quantity and quality. Three-step qPCR was used to evaluate house-keeping and target genes. The conditions were 95°C for 15 min, 40 cycles of 95°C for 10 s, 60°C for 20 s, and 72°C for 32 s. In addition, a melting curve analysis was performed to demonstrate the specificity of PCR product as a single peak. Relative quantification of target gene normalized by β-actin was performed by the 2^−ΔΔCt^ method.

**Table 1:** Sequences of primer pairs used in the real-time quantitative PCR.

Target Gene	Full name	Primer Sequences	Accession Number
*β-actin*	Beta-actin	F: 5’- TGGACTCTGGTGATGGTGTGAC -3’	AF057040.1
		R: 5’- GAGGAAGAAGAGGCAGCGGTTC -3’	
*Sod1*	Cu/Zn-superoxide dismutase	F: 5’- GTCGTCTGGCTTGTGGAGTG -3’	Y12236
		R: 5’- TGTCAGCGGGCTAGTGCTT -3’	
*cat*	Catalase	F: 5’- AGGGCAACTGGGATCTTACA -3’	AF170069
		R: 5’- TTTATGGGACCAGACCTTGG -3’	
*Gpx1a*	Glutathione peroxidase	F: 5’- AGATGTCATTCCTGCACACG -3’	AW232474
		R: 5’- AAGGAGAAGCTTCCTCAGCC -3’	
*apoa1a*	apolipoprotein A-la	F: 5’- TGACAACCTGGACGGAACCGACTA -3’	NM131128
		R: 5’- GCTGCTTGGTGTTCTCCATCAACTG -3’	
*alox12*	arachidonate 12-lipoxygenase	F: 5’- CGATCTTCACCAGCACAGCACAACA -3’	NM199618
		R: 5’- TGTCAGGCAGCGTGTCCATAATCAT -3’	
*alox5a*	arachidonate 5-lipoxygenase a	F: 5’- CGAGAGAGGAGCGGTGGACTCATAT -3’	NM001256747
		R: 5’- GTCATCAACCAACCAGCGGAAGCA -3’	
*sdhaf2*	succinate dehydrogenase complex assembly factor 2	F: 5’- TGCTCCAGAACCGACCATCCTTGA -3’	NM001082864
		R: 5’- TGCGGCTCTCGTACAGCAGTCT -3’	
*gapdh*	glyceraldehyde-3-phosphate dehydrogenase	F: 5’- GACGCTGGTGCTGGTATTGCTCTC -3’	NM001115114
		R: 5’- CCATCAGGTCACATACACGGTTGCT -3’	
*Ldha*	lactate dehydrogenase A4	F: 5’- TGCTCGTTTCCGCTACTTGATGGG -3’	NM131246
		R: 5’- ACGCTCTTCCAGTCCTCCTTGTCTT -3’	
*hemk1*	HemK methyltransferase family member 1	F: 5’- TGCGGTTGTTGTGCTGTGGTAGT -3’	NM001114419
		R: 5’- GATGCGGTGCAGGCTGAAGTGT -3’	
*comta*	catechol-O-methyltransferase a	F: 5’- TGTTGGCATCTGTCCTGGTACTCCT -3’	NM001030157
		R: 5’- CGCTGTGGTCGTGATAGTCCTGTG -3’	
*cyp2aa4*	cytochrome P450, family 2, subfamily AA, polypeptide 4	F: 5’- GCATCGTGGGTATAGTCCGCTATCC -3’	NM001002092
		R: 5’- CGCTCAACGGCTGTGCTGTTATTG -3’	
*cyp2k6*	cytochrome P450, family 2, subfamily K, polypeptide 6	F: 5’- ACGCAGGGTTTGCATTGGAGAGAG -3’	NM200509
		R: 5’- CAGTTGGTGTGGCTTCGGATTCAGT -3’	
*cyp19a1b*	cytochrome P450, family 19, subfamily A, polypeptide 1b	F: 5’- TCCGCTGTGTACCATGTCCTGAAGA -3’	NM131642
		R: 5’- CTGACTTCTGGAGACCTGGACCTGT -3’	

### Statistical analysis

The EF was evaluated to express the enantiomeric compositions [30] and was defined as the enantiomeric concentration ratio (+)/[(-)+(+)] for PCB149. The EF value was in the range of 0 to 1, with the racemate represented as 0.5. The BCF (bioconcentration factor) for aquatic species according to OECD305 was defined as the ratio between the concentration in fish and that in the surrounding media at steady state.

Statistical analyses were performed using SPSS16.0 software. Differences were determined by one-way ANOVA, followed by a *post hoc* Dunnett test. All data were expressed as mean ± standard error of the mean. P < 0.05 was considered statistically significant. MultiExperiment Viewer (MeV) software (open-source genomic analysis created by the MeV development team) was used to visualize the relative levels of gene transcription in all experimental groups using heat maps, based on the fold ratio data of gene expression. The differences in gene expression induced by three forms of chiral PCB149 were investigated by partial least squares discriminant analysis (PLS-DA) and the variable importance (VIP) value in SIMCA-P+11 software (Umetrics, Sweden).

## Results

### Method validation

Satisfactory recovery rates of 97–104% with a relative standard deviation of 2.3–10% were obtained at three spiked concentration levels (0.4 ng/L, 0.1 μg/L, 5 μg/L) in water. As shown in previous study [31], the actual concentrations of chiral PCB149 were less than 20% of the theoretical concentrations for all test periods and no isomerization was observed for either of the two isomers in water. Therefore, the theoretical concentration appropriately represented the actual concentration during the exposure experiments. For embryo/larvae, the spiked concentration were 50 μg/kg, 1.25 mg/kg and 25 mg/kg, and the recovery was in range of 95–110% with a relative standard deviation of 9.8–15%. The concentration of PCB in fish was quantified with standard curve in the range of 5μg/L-1mg/L for (-)- and (+)- PCB149, respectively. The limits of quantitation both for (-)- and (+)- PCB149 in fish were 2.5μg/kg. These recovery rates and their standard errors within 20% were acceptable.

### Non-racemic enrichment

**Fig 1:**
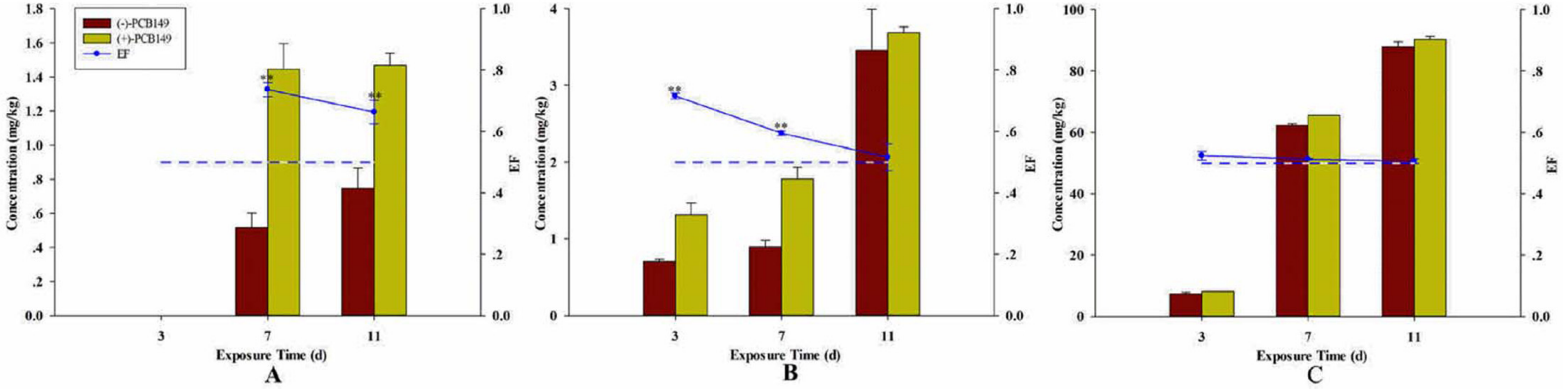
Concentrations of (-)/(+)- PCB149 and EF values within cultured embryo-larvae when exposed to racemic PCB149. A: embryo-larvae exposed to 0.5 ng/L; B: embryo-larvae exposed to 0.1 μg/L; C: embryo-larvae exposed to 2.5 μg/L. Asterisks denote significant difference between treatments and control (determined by Dunnett post hoc comparison, p<0.01, **). Error bars indicate standard deviation.

Stereoselective enrichment was observed when embryos were exposed to racemic PCB149 (Fig 1). Similarly, the concentrations of (-)- and (+)-PCB149 were analyzed and BCF values were also calculated when embryos were exposed to either (-)-or (+)-PCB149 (Table 2). As shown in Fig 1, PCB149 concentration increased with increased exposure time. The concentration after 11 dpe increased to 88.0 mg/kg for (-)-PCB149. This value was 12.2 times larger than that observed after 3 dpe, at a dose of 2.5 μg/L. For (+)-PCB149, the concentration of 90.3 mg/kg after 11 dpe was 11.0 times than that after 3 dpe. Simultaneously, a significant decrease of the EF value, from 0.74 at 7^th^ dpe to 0.66 at 11^st^ dpe was observed. EF values higher than 0.5 were observed, suggesting that non-racemic enrichment happened at the lowest exposures in early development stage of zebrafish.

**Table 2:** Concentrations and BCFs of (-)-PCB149/(+)-PCB149 in embryo-larvae when exposed to atropisomers.

Concentration	(-)-PCB149				(+)-PCB149			
	Concentration (mg/kg (wet wight))			BCF	Concentration (mg/kg (wet wight))			BCF
	3d	7d	11d		3d	7d	11d	
0.5 ng/L	0.9±0.2	1.5±0.1	2.0±0.1	6.6	0.5±0.107	0.9±0.1	1.6±0.1	6.5
0.1μg/L	2.8±0.4	5.2±0.1	5.6±0.2	4.8	1.00±0.1	3.5±0.3	6.4±0.6	4.8
2.5μg /L	12±0.7	146±5.3	133±2.8	4.7	3.5±0.5	91±4.2	115±4.4	4.7

### Toxic effects

The lethal effects and toxic effects such as hatching rate, spontaneous movements and heartbeat of embryos, malformation of embryo and larvae, were observed during the experiments. But the viability and health of embryo and larvae after PCB149 exposure were normal (not shown), which was due to the exposure concentration related to environment was relative low and the investigated cycle was relative short.

### Gene expression

The effect of PCB149 on transcription of genes related to the antioxidant system (*sod1*, *cat* and *Gpx1a*), and the lipid-peroxidative pathway (*apoa1a*, *alox12* and *alox5a*) were examined in our study. Transcription of general metabolic genes related to PCBs metabolism including *cyp2k6*, *cyp2aa4*, *cyp19a1b*, *hemk1* and *comta*, as well as *sdhaf2*, *gapdh* and *ldha*.

**Fig 2:**
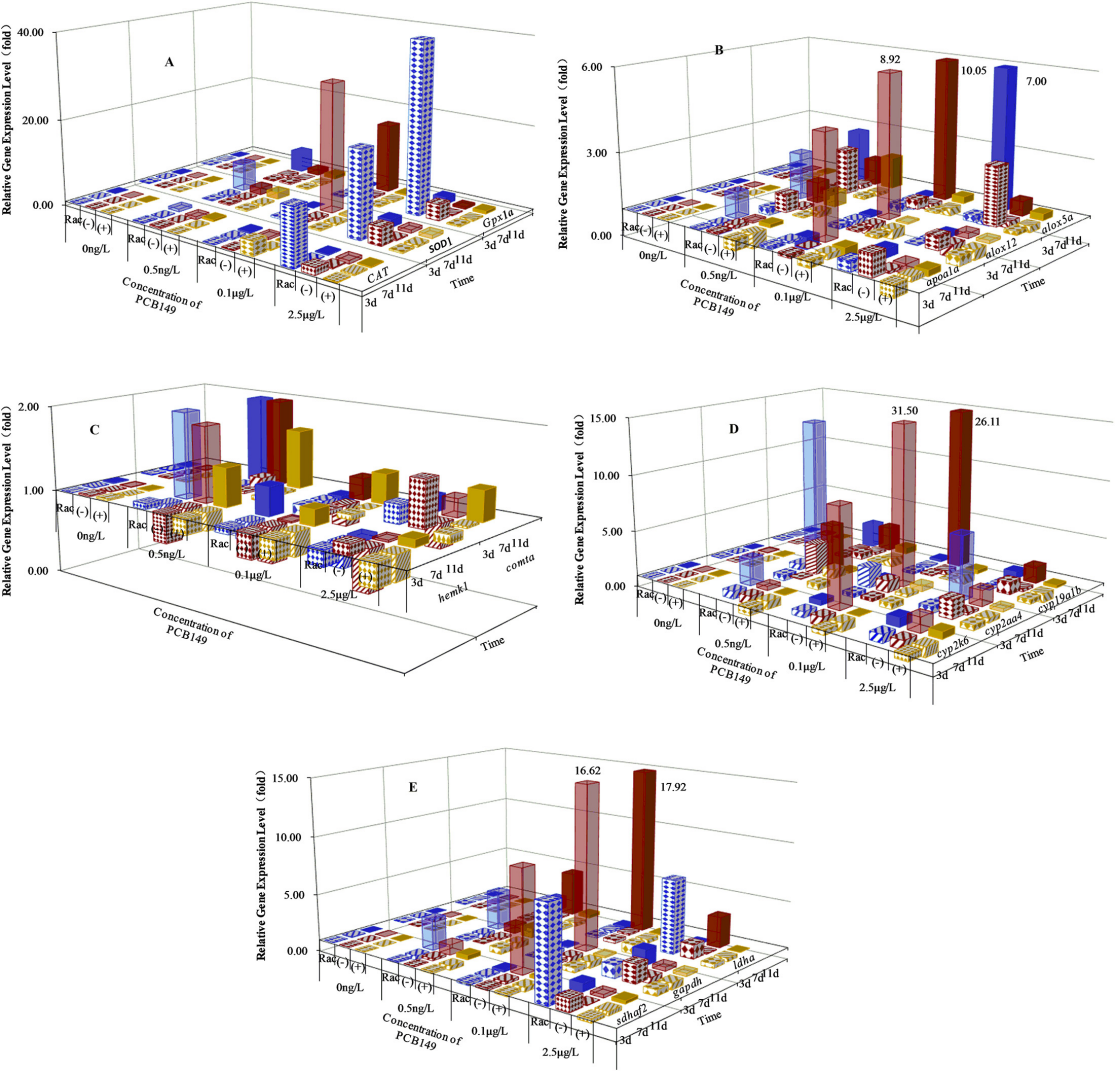
The expression level of indicated genes relative to the no-addition control. A: transcription of genes involved in antioxidant system; B: transcription of genes involved in lipid-peroxidation; C: transcription of genes involved in methylation; D: transcription of CYP450; E: transcription of genes involved in dehydrogenation.

The expression of the *cat*, *sod1*, and *Gpx1a* genes was induced at the highest exposure of the racemic mixture after 3 dpe (Fig 2A). Significant elevation of mRNA expression was observed for both racemate and (-)-PCB149. Exposure to (+)-PCB149 did not induce any significant effects compared with the zero exposure controls. However, the extent of up-regulation caused by the racemate and (-)-PCB149 differed. Elevation of *cat* (by 20.45 fold), *sod1* (by 13.58 fold) and *Gpx1a* (by 179.75 fold) transcription levels were observed after exposure to 2.5 μg/L of racemic PCB149 after 3dpe. Embryo-larvae exposed to (-)-PCB149 at 2.5 μg/L, after 3dpe showed elevated expression of *cat* (by 2.56 fold), *sod1* (by 5.04 fold) and *Gpx1a* (by 4.64 fold) gene. With prolonged exposure to (-)-PCB149, significant inhibition of *cat* expression was found at all three exposure concentrations. In addition, stereoselective induction of genes related to lipid-peroxidation (*apoa1a*, *alox12* and *alox5a*) in embryo-larvae was observed after 3 days exposure to the highest level of racemate (Fig 2B). Maximal induction of these genes was also observed after exposure to (-)-PCB149 at 0.1 μg/L for 11 days.

The *hemk1* gene was induced at the lower concentrations of racemate and (+)-PCB149 in a time dependent manner; however this gene was suppressed at the highest exposure level (Fig 2C). For instance, *hemk1* expression was 1.45 fold at 0.5 ng/L, 1.18 fold at 0.1 μg/L and 1.08 fold at 2.5 μg/L for (+)-PCB149 after 11 dpe. In contrast exposure to (-)-PCB149 generally inhibited expression of this gene. Interestingly, when (+)-PCB149 and (-)-PCB149 existed together as racemate for 11 d, the *hemk1* expression was induced 2.06 fold at 0.5 ng/L, 1.33 fold at 0.1 μg/L and 0.95 fold at 2.5 μg/L. The induction of the *comta* gene was similar to that of the *hemk1* gene.

There was no induction of mRNA expression of *cyp2k6* after 3 days exposure to all three concentrations of racemate or (-)-PCB149 in embryo-larvae (Fig 2D). When exposure time was prolonged to 7 days, significant inhibition of *cyp2k6* expression (0.15 ~ 0.49 fold) was observed. After 11 days exposure *cyp2k6* expression was induced by these compounds. The (+)-PCB149 had little effect. Stereoselective regulation of *cyp2k6* by the (-)-PCB149 was also significantly observed at three different concentrations after 11 dpe. Levels of (-)-PCB149 appeared to account for the regulation of *cyp2k6* expression. The (-)-PCB149 also induced expression of the *cyp2aa4* gene after 11 days exposure. Dysregulation of gene expression related to dehydrogenase genes (*sdhaf2*, *gapdh* and *ldha*) were generally observed in embryo-larvae by the racemate and (-)-PCB149 (Fig 2E).

### Chemometrics

**Fig 3:**
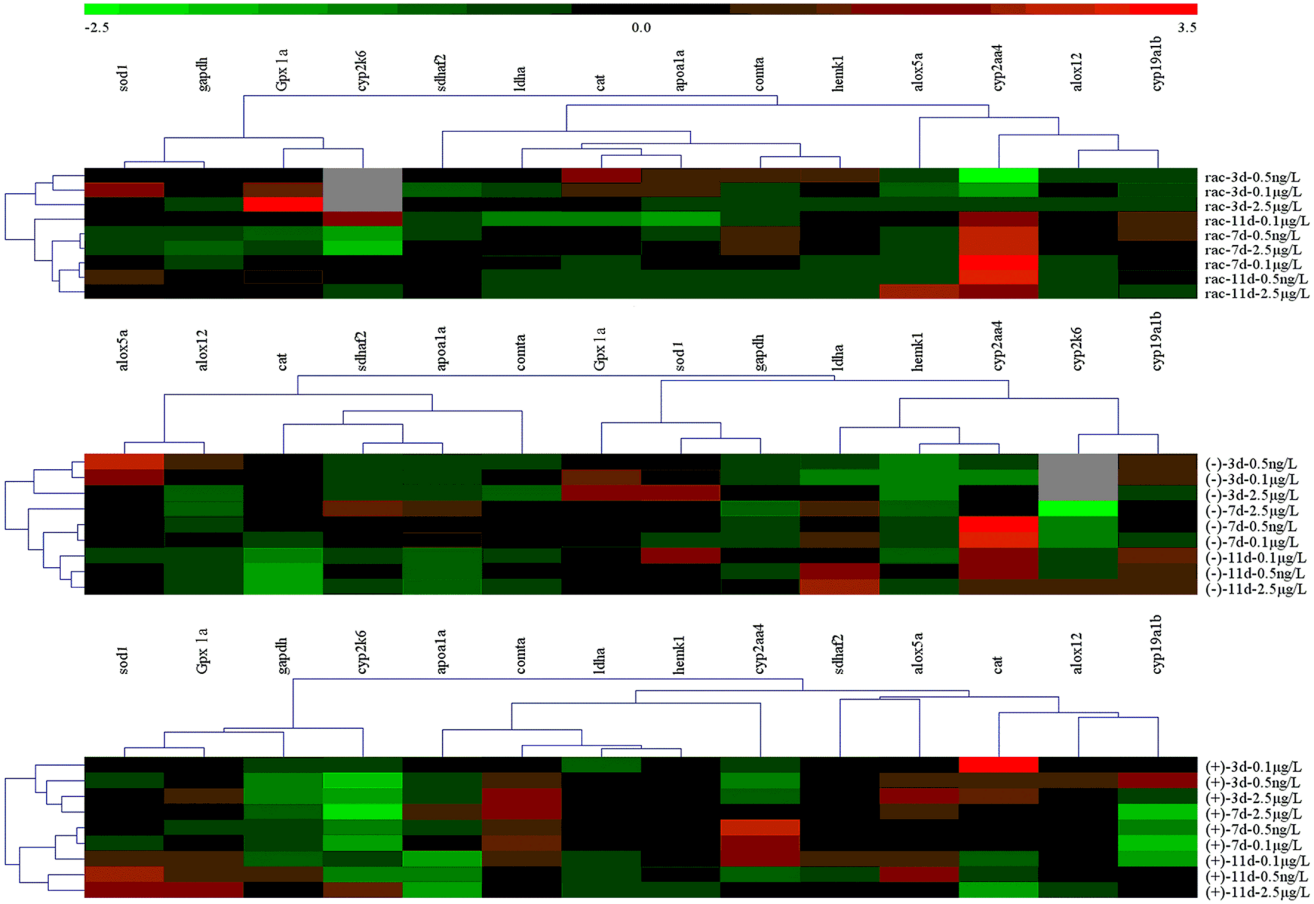
Heatmap of the expression level of indicated genes relative to the no-addition control. Hierarchical clustering was performed and is represented graphically by a color gradient, where red represents the highest level of relative inhibition and green the highest level of relative activation.

**Fig 4:**
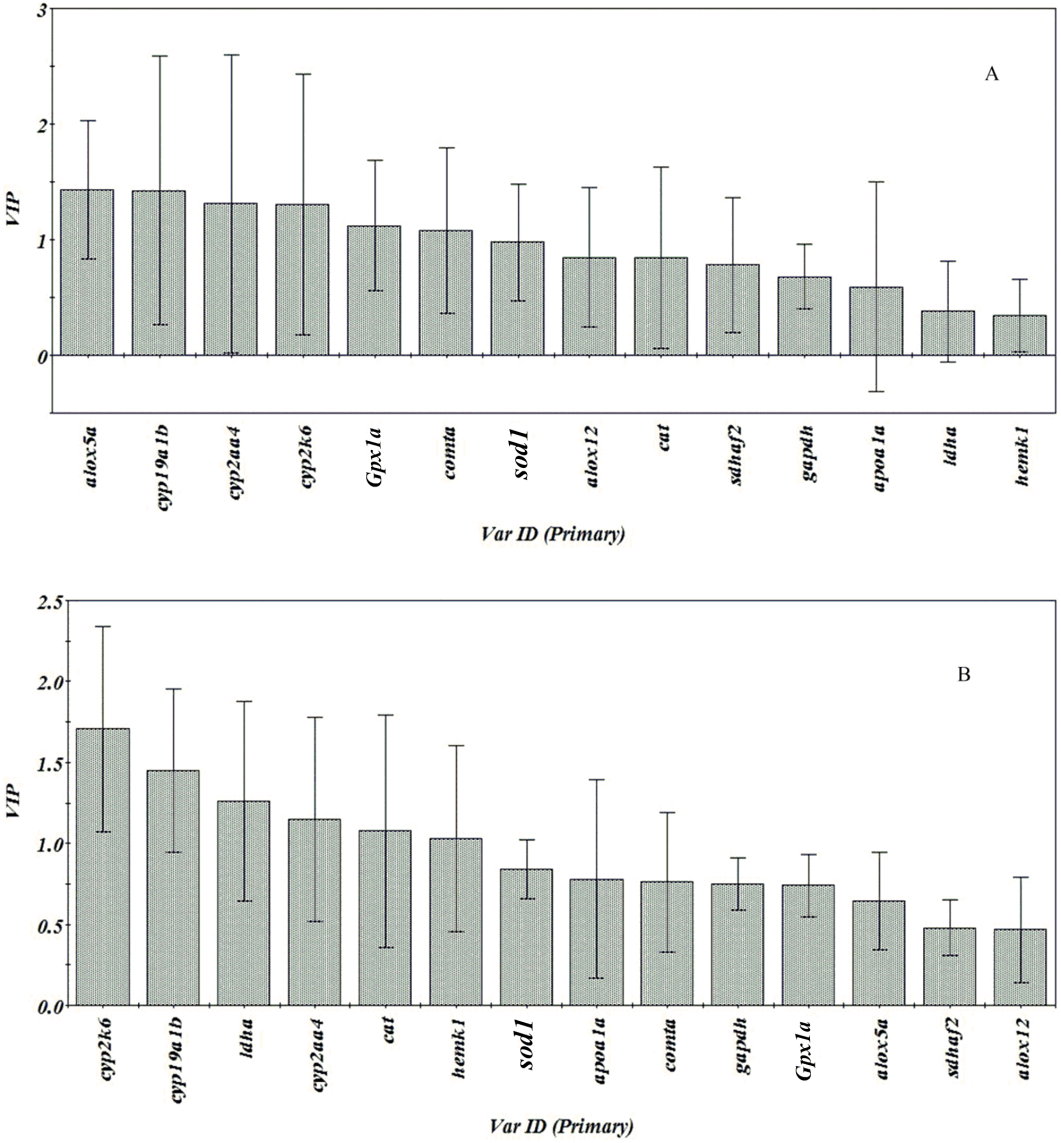
VIP-plot via PLS-DA on the expression level of indicated genes. A: differential genes through comparison of racemic and (+)- PCB149; B: differential genes through comparison of (-)- and (+)- PCB149.

The normalized expression of genes were analyzed through hierarchical clustering based on Pearson correlation coefficients. As can be observed in Fig 3, genes were well categorized in the tree, and different variation tendencies across different groups were observed. In order to better understand the differences of test gene expression induced by three forms of chiral PCB149, VIP plots were given in Fig 4.

## Discussion

In previous studies of chiral PCB149, stereoselective capacity to bioprocess was observed in other adult species. For example, more enrichment of (+)-PCB149 was found in water snakes and barn swallows [32], and an opposite trend was found in mullet samples [33]. However, little reports were related to the species’ developmental stages. EFs of PCB149 congeners were quantifiable in yolk of glaucous gulls’ (*Larus hyperboreus*) eggs from the Norwegian Arctic[34]. A further stereoselective enrichment for PCB149 was observed in the development from eggs to newborn chicks [19].

In this study, experiments of dose-dependent exposure to PCB atropisomers indicated that higher enrichment was found in later embryo-larvae developmental stages. In addition, preferential enrichment of (+)-PCB149 was observed, suggesting that stereoselective accumulation occurred. Racemic enrichment was observed in fish species [12–15], but non-racemic enrichment was observed in embryo-larvae zebrafish. An explanation was that perhaps the stereo-enzyme predominate developmental process during embryo-larvae stage [35]. For the embryo, most of the entire lipid content of the yolk is mobilized and absorbed into the embryonic tissues; and for larvae during the endogenous feeding period, nutrition was provided by the yolk sac before the yolk-sac completely disappeared. This endogenous feeding and a fully developed metabolic pathway [36] may have enhanced enrichment of (+)-PCB149 at this stage.

Based on (+) or (-)-PCB149 exposure experiments, there was no isomerization between the two isomers in our study. The concentration in embryo-larvae exposed to 0.1μg/L atropisomers were only 2–3 times higher than that exposed to 0.5ng/L. In contrast, the concentration in embryo-larvae exposed to 2.5μg/L atropisomers were about 25times higher than that exposed to 0.1μg/L. This observation of enrichment in embryo-larvae after (-)-/(+)- PCB149 exposure could be explained by the BCF values of (-)-/(+)- PCB149 in embryo-larvae. The BCFs of atropisomers in embryo-larvae were in range of 4.67–4.81 when the exposure concentration were higher such as at 0.1μg/L and 2.5μg/L. However, when the exposure concentration was as low as 0.5ng/L, the BCFs of (-)-PCB149 and (+)-PCB149 were 6.60 and 6.51 respectively. This suggested that embryo-larvae had higher ability of (-)-/(+)- PCB149 enrichment when the exposure concentration was relatively low.

We also found that the bioconcentration of isomers was different in embryo-larvae after different exposure as follows: (+)-PCB149 showed higher bioconcentration in embryo-larvae after racemic exposure; however, (-)-PCB149 showed higher bioconcentration after single atropisomers’ exposure. This difference might be due to the ability of (-)-/(+)- PCB149 to permeate into the embryo-larvae. The competitive effects might exist between atropisomers when embryo-larvae were exposed to racemic PCB149. In previous study, competitive interaction with 2B1 isozyme were also observed when different congeners such as chiral PCB45, 95 and 132, were incubated with rat cytochrome P-450 (CYP) 2B1 isozyme [37].

Based on the visual-manual column chart such as Fig 2, data-mining was further used to identify differential gene expression in embryo-larvae induced by racemic, (-)-, and (+)- PCB149. Herein, multiple pattern recognition methods were employed to phenotype the differences between three forms of chiral PCB149. A heatmap representation in our study was used to better understand the classification of target genes. As depicted in Fig 3, we can observe that: (a): genes with similar function was classified to different branches. *sod1* and *Gpx1a* genes were presented as one branch; while *cat* gene was isolated into different branches in treated groups, which suggested that different genes with similar function were contributed to different process. (b): although the gene functions were different, the similar dysregulation were observed in treated groups. The dysregulation of *comta* and *hemk1* genes relevant to methylation were similar to that of *sdhaf2* and *ldha* relevant to dehydration process in racemic treated groups, which suggested that methylation progress had a relationship with dehydration process when embryo-larvae were exposed to racemate. Our heatmap provide an overview of the different classes of target genes and their connection in embryo-larvae exposed to different forms of PCB149. These results in turn help to serve as a guide to understand differences in different forms of PCB149.

Supervised PLS-DA is a better approach for discriminating the constituents in biological samples among the groups. R^2^ (proportion of explained variance), and Q^2^ (proportion of predicted variance) values were used to further evaluate model quality. In general, an R^2^ ≥ 0.65 and Q^2^ ≥ 0.5 indicate satisfactory quantitative predictive ability[38]. In our study, R^2^ and Q^2^ were 0.756 and 0.734, respectively, for which tested gene expression data of embryo-larvae induced by racemic and (+)- PCB149 for PLS. R^2^ and Q^2^ from data induced by (-)- and (+)- PCB149 were 0.872 and 0.798. The above results indicated that the model of PLS is of higher quality. However, the prediction model of PLS was not suitable for tested gene expression data of embryo-larvae induced by racemic and (-)- PCB149.

A VIP-plot loading from PLS-DA clearly displays the leading contributing markers that differentiate the two samples groups [39]. In general, compounds with a VIP score larger than 1 are considered as the important compounds that are the most relevant for explaining the response variable [40]. Thus, the target genes with VIP score, which were present in racemic and (+)- PCB149, (-)- PCB149 and (+)- PCB149 treated groups, were extracted easily by the VIP-plot. As shown in Fig 4A, six test genes (*alox5a*, *cyp19a1b*, *cyp2aa4*, *cyp2k6*, *Gpx1a* and *comta*) were extracted as differential genes for samples induced by racemic and (+)- PCB149. Meanwhile, six test genes (*cyp2k6*, *cyp19a1b*, *ldha*, *cyp2aa4*, *cat* and *hemk1*) were also extracted as differential genes for samples induced by (-)- PCB149 and (+)- PCB149 (Fig 4B). CYP450 enzymes were encoded by a large number of CYP gene superfamily and are involved in the detoxification process when exogenous chemicals enter into an organism such as chemical carcinogens and environmental pollutants[41]. cDNAs encoding CYP2K were isolated from zebrafish in 2005 and CYP2K6 catalyzes the oxidation of lauric acid, which is responsible for fatty acid oxidation in zebrafish [42]. The novel subfamily CYP2AA for zebrafish was found as fish CYP2B-like genes [43]. Enzymes or RNA expression for CYPs related to CYP2B are involved in detoxification processes in mammals [44], but have been inconclusive in fish[43]. *Cyp19a1b* is generally named *cyp19b* and CYP19B protein has been demonstrated as a component of the catalytic complex (nicotinamide adenine dinucleotide phosphate (NADPH)-cytochrome P450 reductase) required for substrate oxidation in bovine [45]. In our study, abnormal expression of CYPs in embryo-larvae after PCB149 exposure suggested that PCB149 exposure could result in the dysfunction of detoxification. CYPs genes (*cyp2k6*, *cyp19a1b*, and *cyp2aa4*) acted as differential genes for (+)-PCB149 exposure, compared with racemic and (-)-PCB149. This results indicated that (+)-PCB149 exposure could be obviously involved in the oxidative processes in embryo-larvae at gene expression levels.

Previous studies also demonstrated that PCBs could cause oxidative stress reaction and then induce abnormal expression of antioxidant genes. This was observed with PCB126 (3,3',4,4',5-pentachlorobiphenyl) [46] in embryonic zebrafish and Aroclor1254 (a highly chlorinated PCB mixture) in minnow (*Gobiocypris rarus*) larvae [47]. In our study, this abnormal expression of antioxidant genes such as *sod1* and *Gpx1a*, was also observed in embryo-larvae after PCB149 exposure, which was consistent with above previous studies. In addition, the dysregulation of antioxidant gene expression had shown that the gene expression changes was at a larger extent when exposure to racemic PCB149 containing 1.25 μg/L (-)-PCB149, compared to exposure to 2.5μg/L (-)-PCB149, which suggested that synergistic effects might exist due to the presence of (+)-PCB149 in the racemate. The results shown in Fig 4A and 4B suggested that changes in gene expression (*alox5a*, *Gpx1a*, *comta* (Fig 4A) and *ldha*, *cat*, *hemk1* (Fig 4B)) from racemic exposure might originate from the influence of (-)-PCB149 exposure. According to gene expression of *cat* and *Gpx1a*, oxidative stress reaction from racemic PCB149 exposure could be mainly induced by (-)-PCB149. The differential gene expression also suggested that enantioselectivity existed in chiral PCB149 at gene transcription levels.

In our study, gene expression of *comta* and *hemk1* relevant to methylation in embryo-larvae exposed to racemic PCB149 could mainly be caused by (-)-PCB149. Methylation pattern is determined during embryogenesis and passed over to differentiating cells and tissues. The addition of methyl groups to DNA could alter the targeting and timing of gene expression and activity of certain enzyme and protein methylation could affect various important cellular process [48]. Three target genes related to dehydration process (*sdhaf2*, *gapdh* and *ldha*) were investigated. The dysregulated expression *of ldha* in embryo-larvae exposed to racemate were related to (-)-PCB149. The enzyme LDHA is a key metabolic enzyme in aerobic glycolysis and considered as a tumor promoter. Dysregulated expression of *ldha* had also been found in squamous cell carcinoma, endometrial cancer cells, and breast cancer cells [49]. Thus, dysregulated expression *of ldha* in our study could potentially accelerate progress in LDHA-targeted cancer therapy. In our study, dysregulated expression of *alox5a* was found in embryo-larvae exposed to chiral PCB149. Dysregulated expression *of ldha* in embryo-larvae exposed to racemate were induced by (-)-PCB149. The enzyme ALOX5 is the key enzyme in leukotriene biosynthesis and expression of alox5 can mainly be found in the myeloid lineage of hematopoietic cells, related to the immune system [50]. Thus, exposure to racemic PCB149 could cause immune-related disease and this could mainly be caused by (-)-PCB149.

## Conclusion

Our results had shown that chiral PCB had stereoselective toxicity to the developmental stage of zebrafish. Based on the bioconcentration analysis, the non-racemic enrichment tended to racemic enrichment, due to a higher enrichment rate of (-)-PCB149 than (+)-PCB149. The stereoselective dysregulation of gene expression and statistical analysis indicated that the effects of (-)-PCB149 was higher than that of (+)-PCB149 to the developmental zebrafish during racemic exposure. Thus, results in our study could assist in elucidating the stereoselective toxicological mechanism of chiral PCB149 during the developmental stages of zebrafish and properly assess PCB risk as an environmental containment.
